# Up-regulated serum lactate dehydrogenase could become a poor prognostic marker in patients with bladder cancer by an evidence-based analysis of 2,182 patients

**DOI:** 10.3389/fonc.2023.1233620

**Published:** 2023-08-03

**Authors:** Xiaoyu Wei, Yumeng Chai, Zhouyue Li, Xuanyan Che, Yong Zhang, Zhongbao Zhou, Xiang Wang

**Affiliations:** ^1^ Department of Oncology, Tianjin Binhai New Area Hospital of Traditional Chinese Medicine, Tianjin, China; ^2^ Department of Urology, Beijing TianTan Hospital, Capital Medical University, Beijing, China; ^3^ Department of Urology, Tengzhou Central People’s Hospital, Tengzhou, China

**Keywords:** evidence-based analysis, bladder cancer, lactate dehydrogenase, prognosis, overall survival

## Abstract

**Background:**

A growing number of studies have considered serum lactate dehydrogenase (LDH) as an indicator of bladder cancer (BC) prognosis. However, a meta-analysis of the serum LDH’s influence on BC prognosis is still missing.

**Methods:**

PubMed, EMBASE, Web of Science and Cochrane Library were exhaustively searched for studies comparing oncological outcomes between high-LDH and low-LDH patients. Standard cumulative analyses using hazard ratios (HR) with 95% confidence intervals (CI) were performed using Review Manager (version 5.3) for overall survival (OS) in patients with BC.

**Results:**

Six studies involving 2,182 patients were selected according to predefined eligibility criteria. The results showed that serum LDH level was significantly associated with OS (HR = 1.86, 95%CI = 1.54-2.25, p<0.0001) in BC. Sensitivity analysis showed the stability of the results. Subgroup analysis revealed that the levels of serum LDH had a significant impact on the OS of BC patients among different groups including publication time, research country, sample size, tumor stage, LDH cut-off value, therapy and follow-up time (all HR>1 and p<0.05), revealing that the ability of serum LDH is not affected by other factors.

**Conclusion:**

Our findings indicated that a high level of serum LDH was associated with inferior OS in patients with BC. However, caution must be taken before recommendations are given because this interpretation is based upon very few clinical studies and a small sample.

## 1 Introduction

Bladder cancer (BC) is a particularly frequent genitourinary cancer (1, 2). Nearly 70 percent to 80 percent of patients had non-muscle-invasive tumor at the time of diagnosis, which can be effectively treated by transurethral cancer surgical removal (3). Nonetheless, twenty percent to thirty percent of newly identified individuals have muscle-invasive BC, and 30% of non-muscle-invasive BC continues to progress to muscle-invasive tumor (3). Regarding patients of muscle-invasive BC, radical cystectomy plus prolonged pelvic lymph node resection is the most common medical strategy (4). Although undergoing radical surgery, long-term prognosis is poor for these patients, with statistics of approximately fifty percent for 5-year survival (5). Immunotherapy, as a developing treatment method for BC in recent years, has unlimited potential, especially with the latest reports of multiple immune predictive molecules including Siglec15 immunotherapy (6), BCAT2 immunotherapy (7), 5mC regulator-based sub-type system (8) and PLR.GHR based prediction model (9) providing deeper insights into the immune therapy response and precise treatment of BC patients. Because the existing tumor staging system performs poorly, predictive variables are required to properly predict oncological results and efficiently divide patients with BC for perfect care.

The Warburg effect occurs when majority malignant cells use metabolism of glucose *via* glycolysis to create sufficient ATP to drive cancer progression (10). Lactate dehydrogenase (LDH), a type of oxidoreductase involved in glycolysis, can transform pyruvate to lactate with the endpoint of glycolysis; hence, LDH is viewed as a prospective biological target in the growth of novel glycolytic inhibitor utilized in cancer treatment (11, 12). Because of its relationship with cancer metabolic alterations, LDH could serve as a marker of cancer progression (13). Numerous investigations have been performed in the prediction of the prognosis of BC patients using serum LDH, but there was still a lack of evidence from the perspective of evidence-based medicine (EBM). There is an urgent need for clear data establishing the predictive relevance of serum LDH in BC. The purpose of this meta-analysis was to determine the effect of serum LDH level on BC outcomes.

## 2 Methods

### 2.1 Search strategy and eligibility

Two authors performed a systematic search of PubMed, Embase, Web of Science, and the Cochrane Library, from inception up until Apr 2023. The search strategy involved only clinical studies assessing the oncologic impact of serum LDH for BC patients. Search terms included: (“Lactate dehydrogenase”) and (“bladder cancer” or “bladder urothelial carcinoma”). Additional manual searching was conducted for pertinent studies and citations. There were no language restrictions on the included articles. The search strategy was designed according to the Preferred Reporting Items for Systematic Reviews and Meta-analysis (PRISMA) statement and AMSTAR (Assessing the methodological quality of systematic reviews) Guidelines (14, 15).

### 2.2 Study selection criteria

Studies were included, if they met all of the following criteria:

(1) Patients with BC at the time of diagnosis;
(2) Oncologic outcomes included overall survival (OS);
(4) Survival data included hazard ratios (HR) and corresponding 95% confidence intervals (CI), or Kaplan-Meier curves comparing survival between high-LDH and low-LDH patients;
(5) Prospective or retrospective studies analyzing the relationship between serum LDH and BC prognoses.

When multiple studies reported findings based upon an identical study population, only the study with most detailed information was included for analysis. Exclusion criteria: not clinical trials, such as abstract, review, comment, *in vitro* studies, case reports, brief reports, conference posters, or animal experiment.

### 2.3 Systematic review process

**Figure 1:**
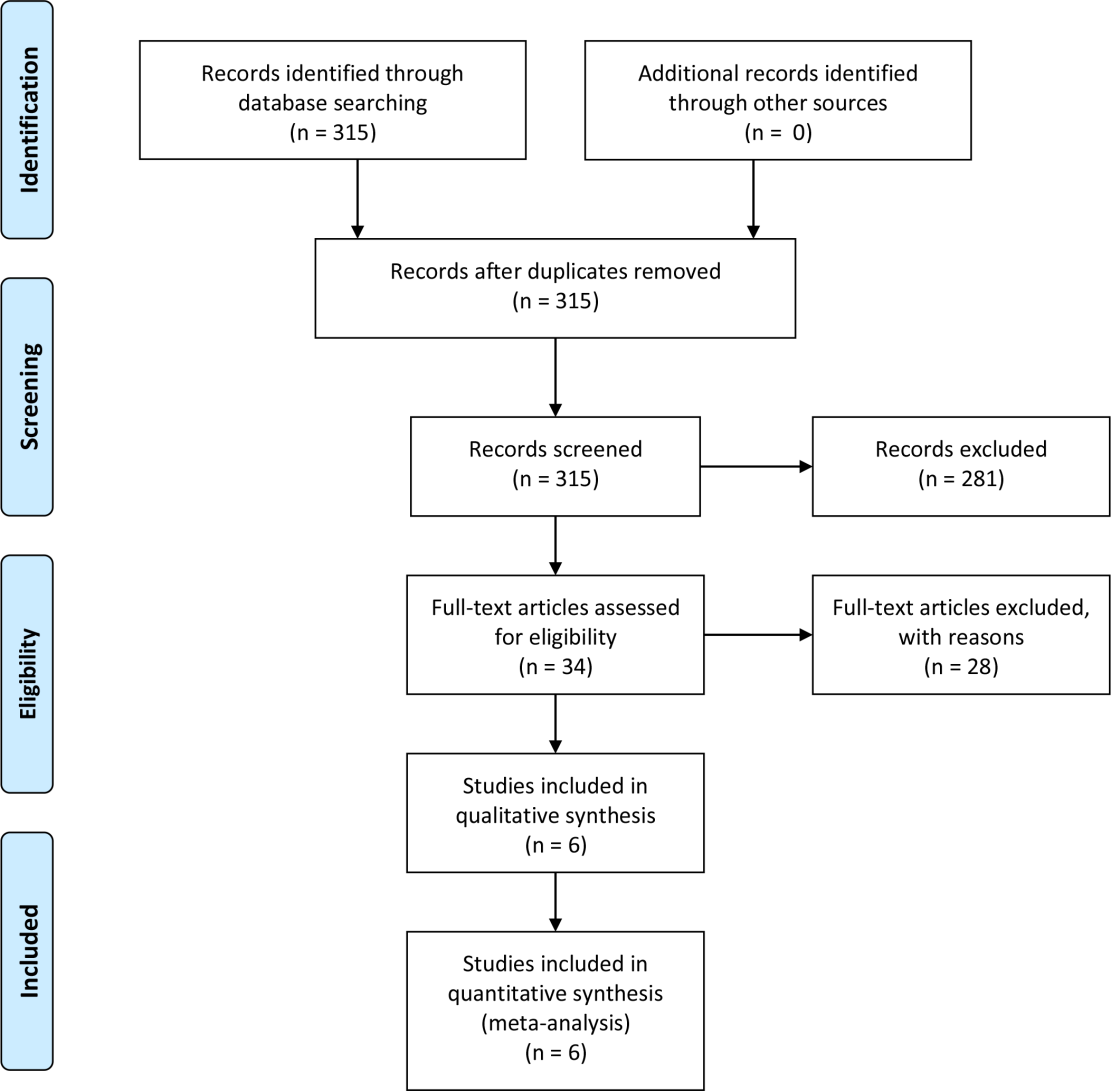
PRISMA of selection process.

After duplicates were removed, two authors performed independent reviews of 315 reports. Discrepancies were resolved through consensus. Eventually, only six studies were selected for data extraction and quality assessment. The PRISMA flowchart depicting the review process is presented in **Figure 1**.

### 2.4 Data extraction

Data were extracted from full length articles by two reviewers independently using a standardized-items form. Extracted information, included: authors, year of publication, country/region, type of study, sample size, age, tumor type, tumor stage, LDH cut-off value, LDH value selection, therapy, survival analysis, outcomes, follow-up time. Hazard ratio (HR) and 95% confidence interval (CI) extraction from multivariate analyses was prioritized. If only Kaplan-Meier curves were available, the relevant data were extracted using Engauge Digitizer 4.1 to calculate HR and 95%CI (16, 17).

### 2.5 Quality assessment and Statistical analysis

The quality of studies was assessed independently by two reviewers using the Newcastle-Ottawa scale (NOS), which is recommended for the assessment of non-randomized studies (18). The NOS scale assesses risk across three domains i.e., patient selection, comparability of groups and outcomes. Any divergences of opinion were settled through discussion or by arbitration with a third reviewer.

Log HR and variance were extracted from all studies and synthesized. For each trial, HRs for survival with corresponding 95% CIs were analyzed in terms of the impact of serum LDH for BC patients on oncologic outcomes. Sensitivity analysis was to be performed where analysis highlighted high levels of heterogeneity. The purpose would be to assess the reliability of findings and to identify potential sources of heterogeneity. Subgroup analysis was used to explore the effect of study classification on the index outcome. All data analyses were carried out with Review Manager (version 5.3; The Nordic Cochrane Centre, Copenhagen, Denmark). A p value of less than 0.05 was considered statistically significant.

## 3 Results

### 3.1 Study characteristics

The search and selection strategy yielded six publications, consisting of six separate clinical studies (3, 11, 19–22). Finally, 2,182 participants were involved and diagnosed with BC. Summaries of demographics, study design and clinical characteristics for each of the included studies has been provided in **Table 1**.

**Table 1:** The characteristics of included studies.

Study	Study design	Coun try	Sample size	Age, years (range)	Tumor	Tumor stage	LDH cut- off value (U/L)	LDH value selection	Therapy	Survival Analysis	Outcome	Follow-up, month (range)
**E. Hannisdal et al.** (20)	Retrospective study	Norw ay	202	73 (32- 85)	BC	Localized	400	–	Radiotherapy	Multivariate	OS	18 (NA)
**Yang MH** **et al.** (19)	Retrospective study	China	310	–	BC	Advanced	200	Reported	Surgery and chemotherapy	Univariate	OS; CSS	71 (1-132)
**Tohru Nakagawa et al. (2013)** (22)	Retrospective study	Japan	114	67 (32- 84)	BC	Recurrent	ULN	Normal	All	Multivariate	OS	11.0 (0.2- 206.7)
**Tohru Nakagawa et al. (2016)** (21)	Retrospective study	Japan	1087	69 (63- 75)	BC	Recurrent	ULN	Normal	All	Multivariate	OS	6.8 (3.0-15.8)
**Su SQ et al.** (3)	Retrospective study	China	263	63 (54- 69)	BC	Advanced	ULN	Normal	All	Multivariate	OS; CSS	34.2 (22.9- 45.8)
**Gu S et al.** (11)	Retrospective study	China	206	–	BC	Localized; Advanced	ULN	Normal	All	Multivariate	OS	48 (NA)

BC, bladder cancer; LDH, lactate dehydrogenase; ULN, upper limit of normal; OS, overall survival; CSS, cancer specific survival; NA, not available.

All included studies’ design type were retrospective study. The studies were conducted in China (n=3), Japan (n=2) and Norway (n=1). The largest study was conducted in China and had 206 participants; the study with the largest number of participants was conducted in Japan, which had 1087 patients. Ages ranged from between 32-85 years and mean age ranged from between 63-73 years. The types of tumor staging included in the study mainly include: localized (n=1); advanced (n=2); recurrent (n=2); localized and advanced (n=1).

Overall, the median follow-up for this sample ranged from 6.8 to 71 months. No significant differences were observed within this sample. All studies were retrospective and after assessment each were found to have obtained scores of ≥6. This level of evidence is broadly considered adequate for meta-analysis (**Table 2**).

**Table 2:** Quality assessment of the included studies.

Study	Selection				Comparability	Exposure			Score
	Definition adequate	Represent of cases	Selection of Controls	Definition of Controls		Ascertainment of exposure	Same method of ascertainment	Non-Response rate	
**E. Hannisdal et al.** (20)	⊕	⊕	◯	⊕	⊕	⊕	◯	⊕	6
**Yang MH et al.** (19)	⊕	⊕	⊕	◯	⊕	⊕	◯	⊕	6
**Tohru Nakagawa et al. (2013)** (22)	⊕	⊕	⊕	⊕	⊕	⊕	⊕	◯	7
**Tohru Nakagawa et al. (2016) (**21**)**	⊕	⊕	⊕	⊕	⊕	⊕	⊕	⊕	8
**Su SQ et al.** (3)	⊕	⊕	⊕	⊕	⊕⊕	⊕	⊕	⊕	9
**Gu S et al.** (11)	⊕	⊕	⊕	⊕	⊕	⊕	⊕	⊕	8

### 3.2 Impact of serum LDH on OS

#### 3.2.1 Overall

**Figure 2:**
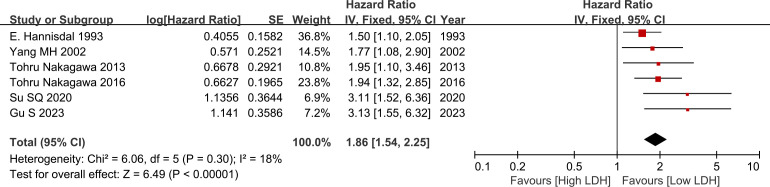
Forest plot of hazard ratio (HR) in BC patients for overall survival (OS).

Pooled analysis suggested that there was no significant difference between the two groups (HR=1.86, 95% CI: 1.54-2.25, p<0.00001, **Figure 2**), low heterogeneity was observed across the trials (I^2 =^ 18%). The results revealed that serum high LDH level was a risk factor for the OS of BC cases (**Figure 2**).

#### 3.2.2 Sensitivity analysis

**Figure 3:**
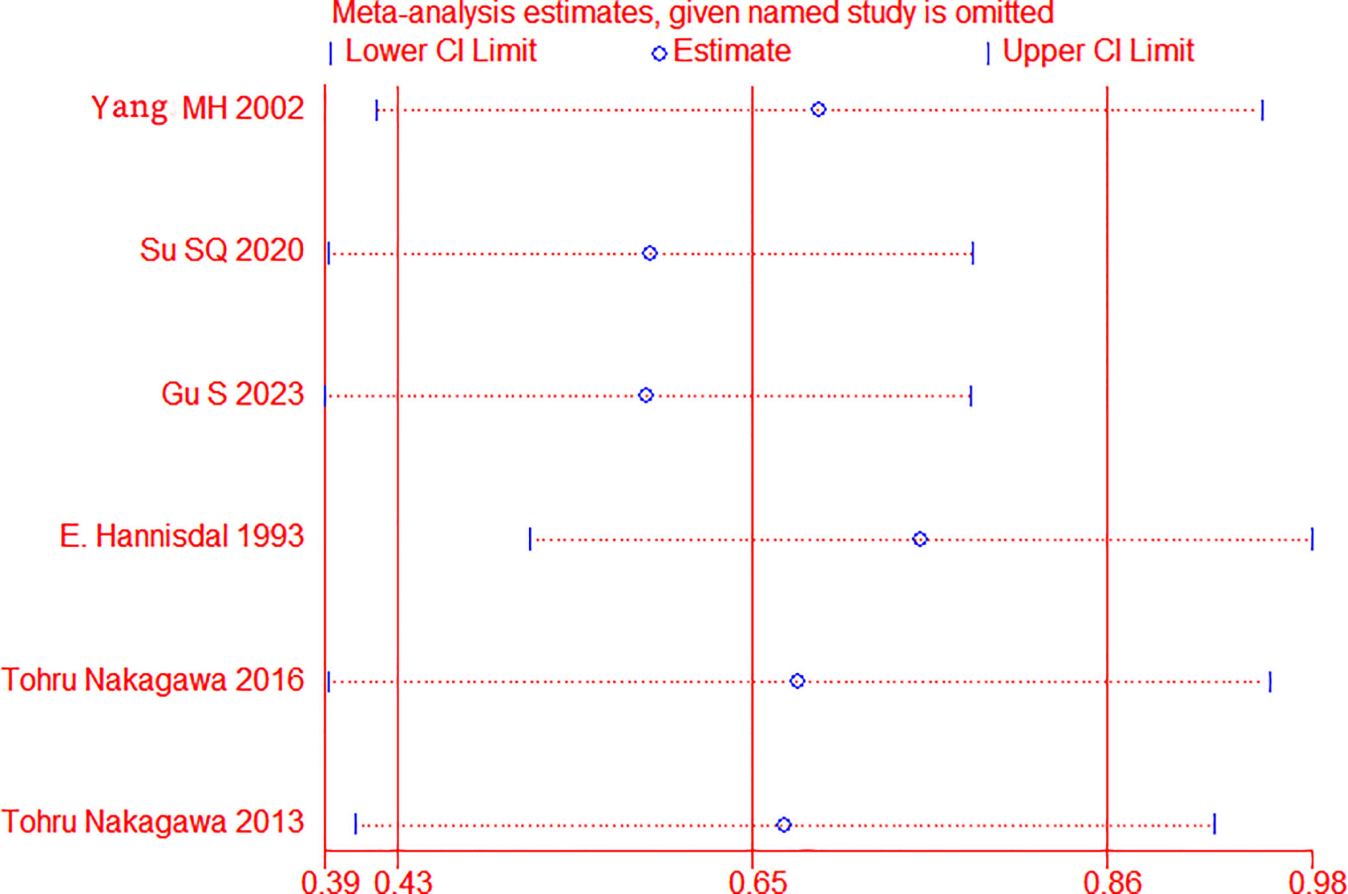
Sensitivity analysis for overall survival (OS) in BC patients.

Sensitivity analysis was applied by removing one trial to assess whether each study significantly affected the pooled HR, see **Figure 3**. The sensitivity analysis indicated that a single study could not significantly alter the pooled results of OS, implying the stability of the results of this study.

#### 3.2.3 Subgroup analysis

In order to explore the factors that may affect the effect of serum LDH on OS in BC patients, we conducted a subgroup analysis, mainly including: Publication time (1993-2015 and 2016-2023); Research country (China and Non-China); Sample size (<250 and ≥250); Tumor stage (Advanced and Recurrent); LDH cut-off value (ULN and Non-ULN); Therapy (All and Non-All); Follow-up time (<24 month and ≥24 month). The analysis found that the levels of serum LDH had a significant impact on the OS of BC patients among different groups, revealing that the ability of serum LDH is not affected by other factors (**Table 3**).

**Table 3:** Subgroup analyses for overall survival in bladder cancer.

Subgroup		Number of studies	HR (95% CI)	P value	Heterogeneity	
					I2 (%)	P value
Publication Time	1993-2015	3	1.63 (1.29, 2.07)	<0.0001	0	0.68
	2016-2023	3	2.31 (1.71, 3.14)	<0.00001	8	0.34
Research Country	China	3	2.34 (1.65, 3.33)	<0.00001	20	0.29
	Non-China	3	1.70 (1.36, 2.12)	<0.00001	0	0.52
Sample Size	<250	3	1.74 (1.35, 2.24)	<0.0001	46	0.16
	≥250	3	2.03 (1.53, 2.68)	<0.00001	0	0.42
Tumor Stage	Advanced	2	2.12 (1.42, 3.19)	0.0003	38	0.20
	Recurrent	2	1.94 (1.41, 2.67)	<0.0001	0	0.99
LDH cut- off value	ULN	4	2.23 (1.70, 2.92)	<0.00001	0	0.49
	Non-ULN	2	1.57 (1.21, 2.04)	0.0007	0	0.58
Therapy	All	4	2.23 (1.70, 2.92)	<0.00001	0	0.49
	Non-All	2	1.57 (1.21, 2.04)	0.0007	0	0.58
Follow-up Time (month)	<24	3	1.70 (1.36, 2.12)	<0.00001	0	0.52
	≥24	3	2.34 (1.65, 3.33)	<0.00001	20	0.29

LDH, lactate dehydrogenase; ULN, upper limit of normal; HR, hazard ratio; CI, confidence interval.

#### 3.2.4 Publication bias

**Figure 4:**
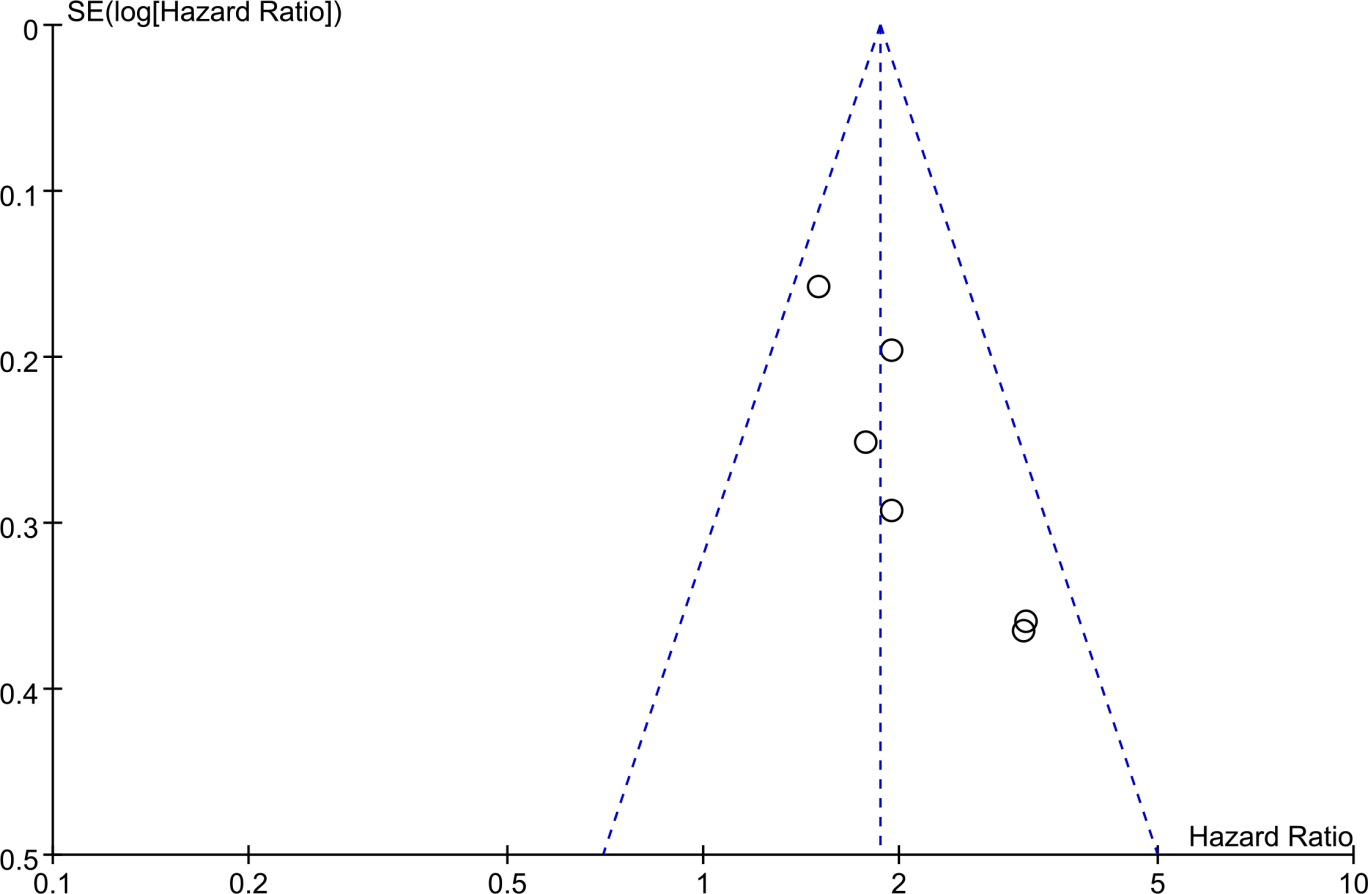
Funnel plot assessing publication bias.

The basic symmetry of the funnel plot indicates that there is no significant publication bias based on this evidence at this stage (**Figure 4**).

## 4 Discussion

This meta-analysis was conducted to investigate the prognostic value of serum LDH in BC. A total of 2,182 patients from 6 eligible retrospective studies were included. The results revealed that serum high LDH level was a risk factor for the OS of BC patients. The sensitivity analysis indicated that a single study could not significantly alter the pooled results of OS, implying the stability of the results of this study. Subgroup analysis revealed that the levels of serum LDH had a significant impact on the OS of BC patients among different groups including publication time, research country, sample size, tumor stage, LDH cut-off value, therapy and follow-up time, revealing that the ability of serum LDH is not affected by other factors.

Earlier studies have discovered that tumor cells metabolism variously than normal ones. Under the presence of enough oxygen, cancer cells would primarily consume glucose *via* glycolysis to create sufficient power for development that is referred to as the Warburg effect and constitutes one of the primary biochemical shifts in the transformation into cancer phase (23). Serum concentrations of LDH, an enzyme engaged in the glycolytic cycle, may indicate changes in metabolism (13). An elevated blood LDH concentration has been demonstrated to function as an adverse predicting indicator for numerous forms of cancer. LDH, a vital part of the Warburg effect, is found in practically all tissues and contains 6 distinct isoenzymes. Most isoenzymes can be made up of two subunits of protein, LDHA and LDHB, that can be arranged into a homo- or hetero-tetramer form (24).

The precise mechanism of predictive relevance of blood LDH in cancer patients is unknown. This was hypothesized that serum LDH levels could indicate the level of hypoxia in tumor cells (3). Due to their fast development, tumor cells are starved of oxygen (3). Cancer cells may deploy anaerobic glycolysis while producing energy, allowing them to be oxygen-independent. LDH is a glycolysis pathway participant that induces the transformation of pyruvate and lactate, and NADH and NAD+ assure the productivity of oncogenic aerobic glycolysis and may be detectable in the blood (25, 26). While tumor tissue necrotizes, excessive cellular amounts of LDH release themselves into the bloodstream, raising serum LDH levels (27). Furthermore, where metastatic spreads arise, cancer cells may cause harm to other tissues including the lung, liver, and bone. The destruction to such tissues may additionally result in a rise in serum LDH concentrations (28, 29). Finally, serum LDH appears to be a crucial determinant in the genesis of cancer. Its concentration can represent tumor load and act as an indication of cancer marker.

Furthermore, higher concentrations of LDH in the blood in cancer patients could represent a sign of immunosuppression. Several investigations have discovered that LDH suppresses and evades the immune system for malignant cells by altering the tumor micro-environment (30). Finally, representing for hypoxia in malignant cells and immunosuppression in malignancy individuals, which suggest a poor prognosis. Furthermore, LDH has emerged as an intriguing biological target for glycolytic inhibitors in potential anti-tumor treatment (31). As a result, serum LDH may be a viable predictive indicator as well as a molecular treatment option for BC. One meta-analysis has previously focused on the predictive importance of preoperative LDH in patients in urothelial carcinoma (UC). Wu et al. published a meta-analysis in 2020 to assess the predictive value of LDH in instances of urological malignancy, finding that an elevated level of preoperative serum LDH correlated with worse OS, CSS, and DFS among individuals with UC (32). Ultimately, serum LDH appears to serve as a crucial determinant in the genesis of cancer. Its concentration can represent tumor load and act as an indication of cancer marker.

While the meta-analysis revealed a strong link between LDH and BC, multiple drawbacks need to be acknowledged. For instance, all of the studies examined used retrospective methodologies, which increases the likelihood of bias. Second, dietary deficiencies, diseases, medications, and lifestyle variables may have altered blood-based markers, leading in biases. Third, no predetermined cut-off ratios for blood-based markers were discovered in the study. Lastly, because the majority of the individuals in our research sample were from an area of Asia, extrapolating the results may be difficult.

## 5 Conclusions

Our findings indicated that a high level of serum LDH was associated with inferior OS in patients with BC. However, caution must be taken before recommendations are given because this interpretation is based upon very few clinical studies and a small sample.

## Data availability statement

The original contributions presented in the study are included in the article/supplementary material. Further inquiries can be directed to the corresponding authors.

## Author contributions

All authors constructed this study. WWe, ZZ, YC, ZL and XC performed the data analysis, figures plotted, and writing. ZZ, XWa and YZ were responsible for the critical reading of the manuscript. All authors contributed to the article and approved the submitted version.
